# Surface Chemistry Study of the Interactions of Sesame Oil with Meibomian Films

**DOI:** 10.3390/molecules27020464

**Published:** 2022-01-11

**Authors:** Petar Eftimov, Norihiko Yokoi, Georgi As. Georgiev

**Affiliations:** 1 Department of Cytology, Histology and Embryology, Faculty of Biology, Sofia University “St. Kliment Ohridski”, 1164 Sofia, Bulgaria; peftimov@uni-sofia.bg; 2 Department of Ophthalmology, Kyoto Prefectural University of Medicine, Kyoto 602-8566, Japan; nyokoi@koto.kpu-m.ac.jp; 3 Faculty of Physics, Sofia University “St. Kliment Ohridski”, 1164 Sofia, Bulgaria

**Keywords:** human meibum, sesame oil, surface films, interfacial properties

## Abstract

A possible approach for the treatment of meibomian gland disease (MGD) can be the supplementation of meibomian gland secretion (MGS) with nonpolar lipids (NPL) rich plant oils. Sesame oil (SO), approximately equal in monounsaturated fat (oleic acid, 40% of total) and polyunsaturated fat (linoleic acid, 42% of total), has shown multiple health benefits due to its anti-inflammatory and antioxidant effects. Thus, the interactions between SO and MGS in surface layers deserve further study. Therefore, pseudobinary films were formed with controlled MGS/SO molar ratios (0%, 10%, 30%, 50%, and 100% SO) at the air/water surface of the Langmuir trough over phosphate buffered saline (pH 7.4) subphase. Surface pressure (π)-area (A) isotherms and Brewster angle microscopy observations showed nonideal interactions where SO aggregates with MGS and complements the NPL stratum of the meibomian layers. The analysis of stress relaxation transients with Kohlrausch–Williams–Watts equation revealed that the supplementation of fixed amount of MGS with excess lipids via SO altered the dilatational elasticity of the films as reflected by the increase of the exponent β. Thus, SO with its unique combination of high oxidative stability and abundance of long polyunsaturated acyl chains might be a useful supplement to MGS layers.

## 1. Introduction

The tear film lipid layer (TFLL) that stabilizes the air/aqueous tear (AT) interface of the tear film (TF) in an open eye, is built primarily (>95%) by the meibomian gland secretion (MGS, or simply meibum) with the putative minor contribution of phospholipids from the lacrimal glands [1,2,3]. MGS is a composite lipid-rich mixture that may also contain up to 22 wt% non-lipid components (proteins, salts, polysaccharides) [3,4,5]. Meibomian lipids consist of >90% non-polar lipids (NPL, primarily wax esters (WE), cholesteryl esters (CE), and triacylglycerides) and <10% polar amphiphilic lipids (primarily (*O*-acyl)-ω-hydroxy fatty acids) [3,5]. When spread over the aqueous tear surface MGS forms a ~100 nm thick viscoelastic duplex film composed of a monomolecular layer of amphiphilic polar lipids at the aqueous interface and an overlaid NPL stratum of unstructured lipophilic suspension facing the air [6,7]. It is now widely accepted that the quantitative or qualitative abnormalities of TFLL, commonly referred to as meibomian gland disease (MGD), are the most common cause of dry eye syndrome (DES), the most prevalent ophthalmic public health disease of modern society, and affect up to 86% of all patients with DES [8,9].

MGD is commonly associated with decreased content of NPL in MGS as well as with altered CE/WE ratio. Thus, a viable approach for the treatment of MGD can be the supplementation of meibum with NPL. A challenge in this direction is that meibomian NPL display a very diverse composition encompassing hundreds of molecular species which essentially makes it impossible to soothe the lipid layer with synthetic lipids. Thus, an alternative approach has gained traction: to utilize GRASE (generally recognized as safe and efficient) plant oils as source of diverse natural NPL. Such strategy was successfully implemented into the Cationorm nanoemulsion where castor oil is used for the purpose. Other plant oil that has shown health benefits [10] is sesame oil (SO). In terms of fats, SO is approximately equal in monounsaturated fat (oleic acid, 40% of total) and polyunsaturated fat (linoleic acid, 42% of total), and the remaining oil content is saturated fat (~8% palmitic acid and 4% stearic acid). This diverse fatty acid composition yields promise that SO can supplement the composite acyl chains profile of human meibum. Furthermore, SO is rich in antioxidants, like vitamin E and phytosterols, which may enhance the resistance of TFLL to oxidative stress thought to play important role for TFLL performance in vivo [11,12,13]. A unique advantage of SO compared to other vegetable oils is that due to its high antioxidant content it combines high oxidative stability with nearly 85% content of unsaturated fatty acid chains [14].

Thus, the interactions between sesame oil and meibomian lipids at the tear-like air/water interface deserve further study as it may provide valuable information for the ophthalmic applications of SO. In order to probe these interactions pseudobinary meibum/sesame oil films were formed with controlled MGS/SO ratios (0%, 10%, 30%, 50%, and 100% SO) at the air/water surface of the Langmuir trough. The study of MGS films with a Langmuir surface balance has proven as an efficient approach to access the surface properties of the sample at physiologically relevant blink-like deformations [2]. Langmuir trough studies were also applied to study the surface behavior of lipid containing eyedrops [15,16]. Surface pressure (π)-area (A) isocycles and stress relaxations were used to assess (i) the layer’s reorganization during area cycling and (ii) dilatational elasticity respectively. Film morphology at the different MGS/SO ratios was monitored by Brewster angle microscopy (BAM).

## 2. Results

### 2.1. Alternating MGS Amount in the Film-Forming Solutions of the MGS/SO Films

The first set of experiments was performed with pseudobinary MGS/SO layers where the decrease of MGS content is compensated with a proportional supplementation of SO in the film-forming solutions. This regime allows to analyze the (non)additivity of MGS/SO interactions.

**Figure 1:**
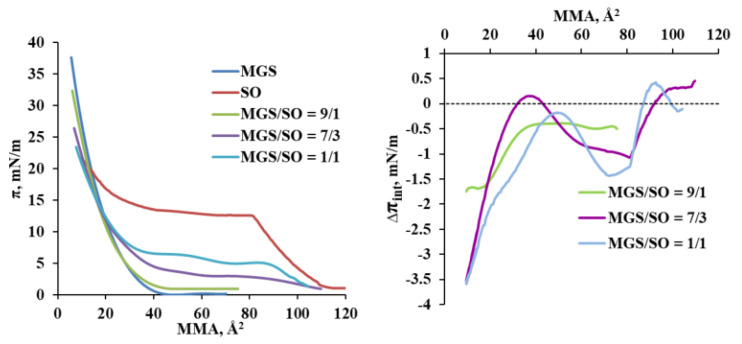
**Left panel:** Surface pressure (π)/area (A) isotherms of MGS/SO pseudobinary films. The decrease of MGS content is compensated by a proportional increase of SO content in the film-forming solutions, so that the isotherms can be analyzed with Equation (1). **Right panel:** analysis of the MGS/SO interactions via application of Equation (1) to the π(A) isotherms of the pseudobinary films. The dashed line corresponds ∆π_int_ = 0, i.e., ideal additive MGS/SO interactions. ∆π_int_ < 0 and ∆π_int_ > 0 show deviations from ideality, which in the context of the multilayer structure of the films indicate (i) aggregation of lipid molecules in thick surface domains or to (ii) the domains disaggregation at the interface respectively (see the main text for details).

The surface pressure/area isotherms of the various compositions are presented at Figure 1, left panel. It can be seen that for pure meibum the onset of the surface pressure took place at apparent molecular area of 45 Å^2^ and at further compression, the surface pressure increased smoothly to 38 mN/m at 10 Å^2^ (the interpretation of molecular areas in MGS containing multilayers is referred in the Discussion section). These data are in agreement with Brewster Angle Microscopy (discussed below) and with many other studies which showed that MGS never exists in a monolayer state [1,2]. Instead, it forms multilayer patches immediately after spreading which transform into a continuous duplex film at further compression. Pure SO film was in monolayer state up to 84 Å^2^ and at further compression a multilayered structure is adopted as reported for other surface films by plant oils and other triacylglycerol rich mixtures [17,18].

The MGS/SO pseudobinary layers similarly to MGS also displayed multilayer structures immediately at their deposition at the air water surface. The surface pressure increments ∆π_int_ were calculated according to Equation (1) which allows to quantify the deflection of the system from ideality [19].

∆π_int_ = π_A, expl_ − π_A, ideal_ (1)

Here

π_A, ideal_ = X_MGS_∙(π_MGS_)_A_ + X_SO_∙(π_SO_)_A_ (2)

where X_MGS_ and X_SO_ are the molar fractions of the two components, and (π_MGS_)_A_ and (π_SO_)_A_ represent the surface pressures of the “pure” components at an area per molecule A.

**Figure 2:**
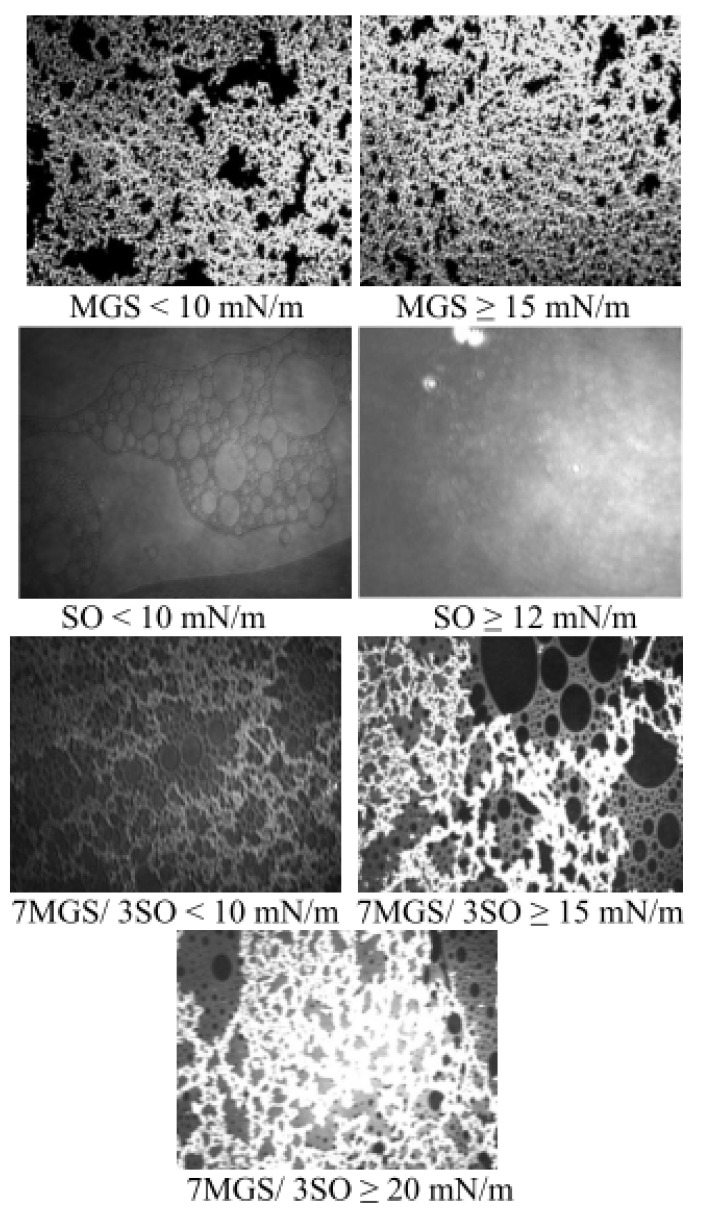
BAM images (500 µm × 300 µm) of MGS, SO and pseudobinary MGS/SO films.

Thus, the interactions between the two components can be quantitatively studied by the surface pressure increment, ∆π_int_, which is calculated as the difference between the ideal surface pressure, π_A_,_ideal_, as expected by the additivity rule and the experimental value, π_A_,_exptl_. Although Equation (1) was first applied to monolayers, the additivity rule remains a valid tool to estimate deviations from ideality also for films that are not in monolayer but in multilayer state and the additivity law stated by Equation (1) was already successfully applied to study the interactions of meibomian multilayers with polar lipids, cationic nanoemulsions, sebum (other inherently multilayer forming physiological lipid mixture), fatty acids etc., as well as interactions in adsorption films of pharmaceutical compounds [20,21,22,23,24]. As explained elsewhere [20,21,22] for films containing multilayer aggregates, Equation (1) did not measure 2D intermolecular attraction or repulsion. Here the quantities indicate more complex transitions related to (i) aggregation of lipid molecules in thick surface domains or to (ii) the domains disaggregation at the interface. Thus:

- ∆π_int_ = 0 denotes ideal behavior where the two compounds do not alter each other’s organization at the air/water surface;
- ∆π_int_ > 0 may report for both, (i) repulsion between the lipid molecules in the plane of the film/water interface and/or (ii) disaggregation of the multilayer domains and redistribution of lipid molecules from the non-polar stratum to the aqueous interface;
- ∆π_int_ < 0 may report for both, (i) aggregation between the lipid molecules in 2D at the film/water interface and/or (ii) increased aggregation of the molecules within the multilayer and redistribution of lipid molecules from the aqueous interface toward the film’s non-polar stratum facing the air. The formation of thicker brighter regions in the MGS/SO films visualized by the Brewster angle microscopy images (Figure 2) suggests that it is the latter phenomenon that takes place.

The ∆π_int_/MMA are shown in Figure 1 (Right panel). It can be seen that while at large MMA, the increment ∆π was ≥0 or just slightly negative, at further compression ∆π values become negative with a steep increase of the absolute value of the negative ∆π increment at ≤50 Å^2^ apparent area per molecule for layers with 10 and 50% SO content and at ≤37 Å^2^/molecule for layers with inclusion of 30% SO. Analysis of the nonideality of MGS/SO interactions was also performed in terms of area changes, ∆A_int_, which revealed similar trends (see Supplementary Materials).

Brewster angle microscopy images also revealed that at surface pressures higher than 12 mN/m increased aggregation is observed in the MGS/SO films with thicker brighter regions formed in the layer that become more prevalent at further compression.

**Figure 3:**
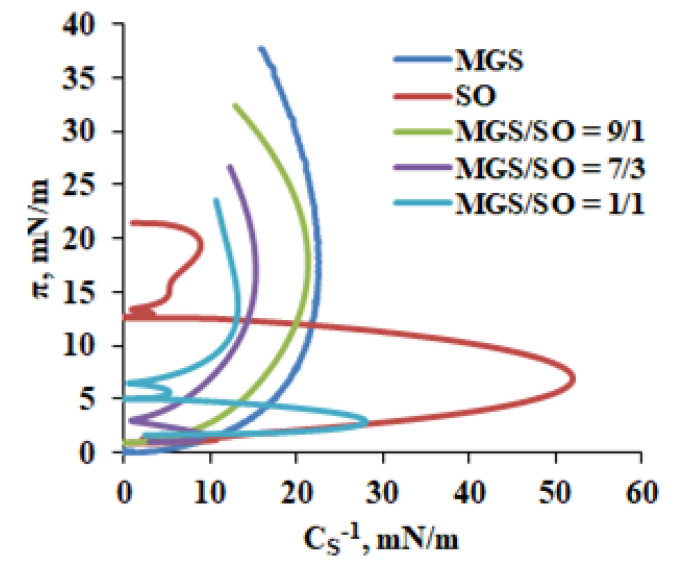
Dependence of the reciprocal compressibility (or rigidity) modulus on the surface pressure of the MGS/SO layers.

The analysis of the reciprocal compressibility (or rigidity) modulus C_s_^−1^ (Figure 3) was done via Equation (3).

C_s_^−1^ = A_π_ (−dπ/dA) (3)

where A_π_ is the area at the indicated π. The inflexion points in the π/Cs^−1^ dependencies indicate the surface pressures at which significant reorganization of the surface film takes place in the course of the film compression. According to the Davies and Rideal [25] criterion, for C_S_^−1^ < 50 mN/m the state of a given monolayer is classified as liquid expanded (LE), for 100 < C_S_^−1^ < 250 mN/m the state is liquid condensed (LC), for C_S_^−1^ > 250 mN/m the monolayer is described as solid state.

### 2.2. Pseudobinary MGS/SO Films with Fixed MGS Amount

**Figure 4:**
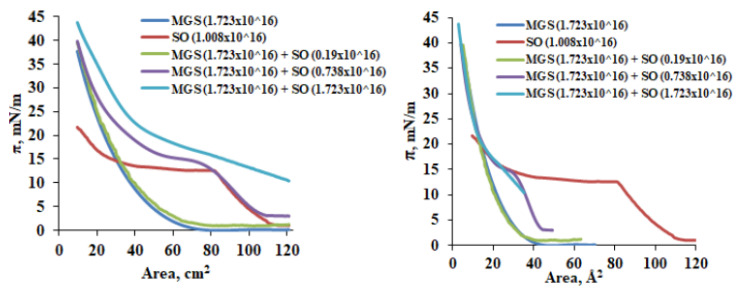
Surface pressure (π)/area (A) isotherms of MGS/SO pseudobinary films presented in terms of total film area (cm^2^; **Left panel**) and apparent area per molecule (Å^2^; **Right panel**). The numbers in brackets denote the total number of MGS and SO molecules deposited at the trough surface. For the interpretation of the values of area per molecule for meibum containing films please refer to the main text. Here MGS content is kept constant and the total lipid amount is increased by supplementation with SO. This regime is similar to the effect of SO delivery via eye drops at the ocular surface (see main text or details).

In this set of experiments, fixed amount of MGS is spread at the air/water surface of the Langmuir trough and SO is overlaid on top of it. Thus, the addition of SO results in increased amount of total lipid at the air/water surface. The results for different compositions are shown in Figure 4. 

This measurement regime emulates the conditions of an in vivo application of SO, where sesame oil will be supplemented to fixed amount of TFLL and will result in an increase of the total lipid concentration at the ocular surface.

It can be seen that the inclusion of extra lipid material via supplementation with SO resulted in (i) an increase of the surface pressure achieved at maximum compression of the surface films, (ii) overall shift of the π(A)-isotherms to higher surface pressures, and (iii) change of the shape of the isotherms to partially resemble the one of pure SO. This is indeed the normal and expected behavior when lipid molecules are supplied in excess per unit area [21] as the increase of the amount of lipids per unit area expectedly raises the surface pressure even prior compression is initiated. This is also the reason the onset of the isotherms at the right panel of Figure 4 is shifted not only to higher surface pressures but also to lower apparent areas per molecule. The latter reflects the growing number of lipid molecules per unit area resulting from the supplementation of SO to the fixed MGS amount.

**Figure 5:**
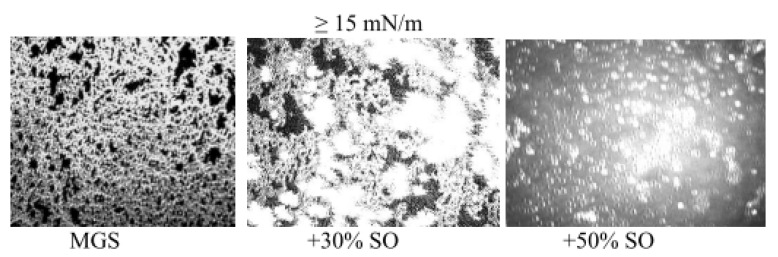
Thickening of MGS films via supplementation with SO (brightness of the images is attenuated to ensure visibility) (micrograph size is 500 µm × 300 µm).

Brewster angle microscopy reveals (Figure 5) that the delivery of extra SO to the meibomian films results in overall thickening of the films (as revealed by the increased brightness of the BAM micrographs) and disappearance of the monolayer patches in the layers in presence of 30% and 50% SO.

**Figure 6:**
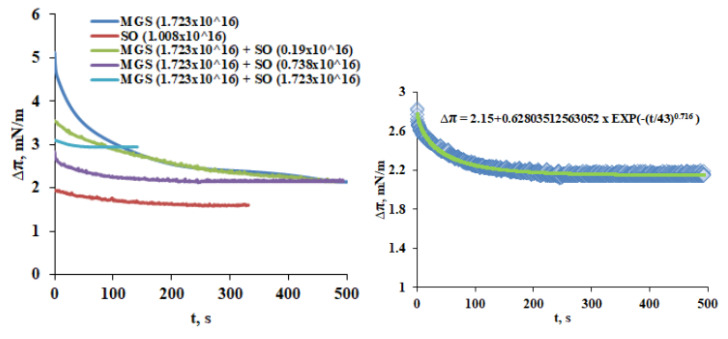
**Right panel**: Stress relaxation transients of MGS films supplemented with extra amounts of SO. The numbers in brackets denote the total number of MGS and SO molecules deposited at the trough surface. **Left panel**: Stress relaxation transient fitted with Kohlrausch–Williams–Watts (KWW) equation (Equation (4)).

Viscoelasticity is considered important for the capability of TFLL to maintain its structure and the overall stability of TF at consecutive blinks. That is why the dilatational viscoelasticity of the layers was probed via the step deformation technique and the surface pressure relaxation transients are presented in Figure 6.

The relaxation transients were analyzed via the Kohlrausch–Williams–Watts (KWW) equation of the form (Equation (4))

Δπ = A. exp (−(t/τ)^β^) + Δπ_EQ_ (4)

Here the value of β is a useful material characteristic that can be utilized to distinguish between samples, as higher β is associated with a greater tendency for viscoplastic deformation responsible for stress decay [26]. Two relaxation regimes are possible with β ≤ 1 (stretched exponential) or β ≥ 1 (compressed exponential). For microscopically heterogeneous systems like the ones studied here the fractional exponent β decreases further away from unity (i) with increasing interaction strength between the units and/or with (ii) increasing the number of interacting arrays (i.e., with increasing the heterogeneity of the material) [27]. Physical interpretation of the values of β was proposed on the base of a spatial rearrangement model [28,29]:

- β = 3/4—self-avoiding walks in 2D;
- β = 3/5—self-avoiding walks in 3D;
- β = 1/2—self-avoiding walks in 4D; Brownian motion; diffusion-limited growth/segregation;
- β = 1/3—anomalous diffusion on percolating clusters; growth of ensemble of clusters—Ostwald ripening; spinodal decomposition;

In the case when β > 1 the so called i.e., compressed exponential function takes place. It is characteristic for very slow dynamics, the so-called jammed state of soft matter in which no fast relaxation channel exists [20]. In Equation (3) Δπ_EQ_ is a measure for the equilibrium elasticity after completion of the relaxation.

It can be seen (Table 1) that for MGS layers β = 0.486. For MGS/SO = 9/1 and 7/3 β grew but still remained < 1, while for SO enriched layers (MGS/SO = 1/1 and pure SO) the layers showed β > 1. Although for the complex compositions used in the study it is not possible to provide a specific explanation for the behavior of β, the result aligns with recent findings that interactions between non-polar lipids can modify the order and the phase transition of the meibomian layers in a nonlinear fashion [30].

## 3. Discussion

Due to the extreme hydrophobicity of its constituents MGS does not spread to a monolayer. Instead, as revealed by Brewster Angle Microscopy, here and in other studies, even at (very) low surface pressures (0.5–10 mN/m) MGS forms heterogeneous continuous duplex films at the air/water interface which get more uniform when π increases and retains liquid extended-like in-plane reciprocal compressibility for the whole π range [1,2]. MGS performance at the air/water surface resembles that of triglyceride/cholesterol ester mixtures (i.e., compositions partially similar to meibum), which also form non-collapsible and reversible multilayer films [31,32]. A key feature of such thick films enriched with hydrophobic lipids (like the wax and sterol esters in MGS) is that at area contraction the polar lipid molecules (located at the film/aqueous interface), instead of being compressed to a condensed state and then forced to collapse (the usual reason for the loss of material at monolayer cycling), migrate to the upper NPL-enriched stratum of the layer [1,2,31]. Then this upper stratum of the film acts as an interfacial reservoir from which polar lipids rapidly spread back on the surface during area expansion. The latter phenomenon results in high stability of the layers at continuous area cycling. The MGS roughness heterogeneity (manifested as brightness heterogeneity across BAM images) suggests that it is not a stacked lamellar structure, but as recently proposed is a duplex film of a polar lipid monolayer with a continuous oily fluid on top of it with some crystallites dispersed within [7]. Thus, the duplex structure renders the MGS film non-collapsible and highly resistant to the loss of material during cycling and might well be responsible for the characteristic surface properties of MGS. Possible in vivo consequences are that the film can maintain its performance at the air/tear surface for an extended period of time and there is no need for the meibomian glands to constantly secrete new lipids at the interface. These assumptions agree well with the in vivo observations that TFLL turnover rate (0.93 ± 0.36%/min) is much slower than the one of the aqueous tear fluid (10.3 ± 3.7%/min) and hence the full exchange of TFLL takes hours to complete [33].

SO films displayed behavior typical for other plants oils with similar compositions [32,34]. Up to 83 Å^2^ and π = 12 mN/m the SO exists in a monolayer form and at subsequent deformation it collapses to a thicker bilayer film. Then the surface pressure remains stable up to 40 Å^2^ when π rises to reach 21.7 mN/m when the compression is completed. Such increase of surface pressure was previously reported for overcompressed layers of triglycerides (~98% of SO composition) and of other hydrophobic lipids like cholesterol and is attributed to the formation of trilayers [35,36,37]. Another contribution to this behavior is the complex composition of sesame oil which consists of triglycerides with different acyl chain profiles: 8% GS2U, 41% GSU2, and 51 % GU3 respectively (here: G—glyceride backbone, U—unsaturated and S—saturated acyl chain respectively) [38]. The plateau at π = 12 mN/m corresponds to the collapse pressure of GU3 (trioloein and trilinoolein) [39]. However GS2U and GSU2 have higher collapse pressures and will therefore require further compression to desorb from the interface into the bulk oily phase overlaid on top [40]. The formation of thick SO films is well confirmed by the BAM micrographs of the layers. The behavior of SO and MGS/SO mixtures at high degree of compression is important as it resembles the extreme area changes observed at the ocular surface at blink.

**Figure 7:**
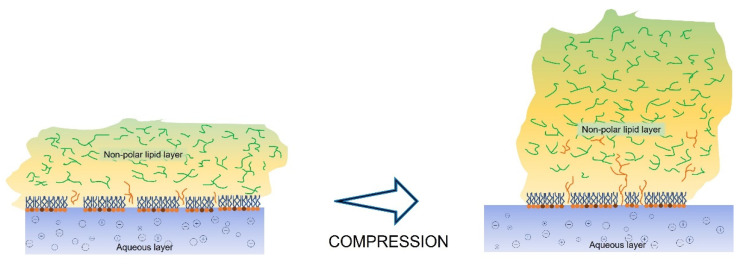
A simplified figure of the redistribution of sesame oil’ GS2U and GS3U (G—glyceride backbone, U—unsaturated and S—saturated acyl chain respectively) triglycerides between the polar and non-polar fractions of the TFLL; compression engender their climbing into the NPL stratum (see main text for details).

The miscibility experiments with MGS/SO pseudobinary layers reveal that at high MMA and low π, the SO molecules can contribute to the molecular packing at the aqueous/lipid interface as manifested by the slightly positive or slightly negative ∆π values at these conditions. However, at further compression ∆π becomes increasingly negative which indicates that large portion of the molecules in the MGS/SO pseudobinary films climbs into the NPL stratum of the layers. The phenomenon is also visualized by BAM images (Figure 2) where the formation of a thicker brighter, presumably SO-enriched, layer can be clearly seen in MGS/SO films. Similar trends are revealed when the film behavior is analyzed via changes in the area increments (∆A_int_; see Supplementary Materials). Considering the high hydrophobicity of SO ingredients and the fact that SO cannot maintain monolayer structure at π ≥ 12 mN/m it can be reasonably assumed that the GSU2 and GU3 triglycerides in sesame oil climb on top of the MGS layer and distribute among the NPL stratum (Figure 7). It is also important to note that for all MGS/SO layers C_s_^−1^ remained lower than 30 mN/m and the shape of the π(C_s_^−1^) dependencies remained similar to the one of pure MGS. Such behavior is further proof that SO has limited contribution to the surfactant layer at the film/aqueous interface and instead contributes primarily to the NPL stratum of the layers.

It should be kept in mind that the interpretation of the mean area per molecule (MMA) value is different for the monolayer and multilayer systems. For sesame oil (SO) up to 84 Å^2^ the film is in monolayer state and the MMA has the standard meaning of a crude averaged estimate of the area occupied by a lipid molecule at the water surface. For meibum (MGS and MGS/SO)-enriched lipid layers, natural and synthetic, the lipid molecules never exist in a monolayer state [1].

Rather due to their extreme hydrophobicity, the lipid molecules are predominantly in the form of multilayer aggregates even at zero surface pressure prior the onset of the surface pressure/area isotherm [41]. Thus, the lift-off of the surface pressure is commonly reported to take place at MMA in the range of 30–60 Å^2^ i.e., well below the molecular areas that can be expected in a monolayer [22,31,34,42]. Thus for MGS-containing layers MMA should be considered as an apparent value that indicates not the space occupied by a molecule at the surface but rather the distribution of the molecules between the aqueous interface and the upper, extended toward the air, non-polar stratum of the meibomian surface film, i.e., 20 Å^2^ apparent area per molecule indicates that if ~ 60 Å^2^ limiting area per molecule is assumed [34] then two-thirds of the molecules in the layer are not located at the film/water interface but are instead distributed in the hydrophobic stratum of the multilayer [2,31,42,43]. Furthermore, in previous studies, ours and by other teams [1,6,7], it was confirmed by analysis of Brewster angle and polarizing microscopy images, as well as X-ray diffraction data, that at ≥15 mN/m the meibomian layers represent thick duplex films with thickness in the range of around 4–6 monolayers. The in vitro data align with the in vivo ones. The thickness of the healthy tear film lipid layer at the ocular surface ranges from 75–170 nm (the thickness of a lipid monolayer is ~2–2.5 nm) in an open eye during the interblink and increases further upon compression by the eyelid at blink [1,2,5].

The assumption of SO being primarily distributed to the NPL stratum of the films is well supported by the studies with increase of total lipid amount via SO supplementation (Figure 4, Figure 5 and Figure 6). The delivery of extra lipid material in the form of SO results in the formation of thick homogeneous duplex films as visualized by BAM which expectedly display higher lift-off surface areas and higher maximum surface pressure achieved at the completion of film compression. Although the extra supplementation of SO had limited effect on the equilibrium elasticity of the layers (with exception of MGS/SO = 1/1, for the rest of the layers ∆π remained about the same at ~2 mN/m) the shape of the relaxations, i.e., the nature of the processes involved, was modified with the power β raising from 0.43 (pure MGS) to β ≥ 1 in the presence of SO. This reflects that the inclusion of SO soothed the inherent heterogeneity of the NPL “cap” of the pure meibomian layer which is also manifested by its heterogeneous BAM appearance and resulted in the formation of homogeneous and tightly packed layer partially similar to the one observed in pure SO multilayer at high compression.

At physiologically relevant packing densities and surface pressures SO complements the composition of the NPL stratum of the meibomian layers and stabilizes their elastic properties. These properties as well as its antioxidant content, oxidative stability, and high content of unsaturated fatty acid chains suggest that SO can be a suitable ingredient in the formulations for the treatment of dry eye as well as for ophthalmic drug delivery [14].

## 4. Materials and Methods

### 4.1. Materials

Human meibum from healthy volunteers was donated by Prof. Douglas Borchman, University of Louisville, Louisville, KY, USA. MGS was collected with permission by the institutional review board, in agreement with the tenets of the Declaration of Helsinki and after the healthy volunteers signed informed consent. Meibum was expressed from the eye lids of each volunteer using an ILUX instrument (Alcon, Fort Worth, TX, USA) according to the manufacturer’s instructions after a mild anesthesia with proparacaine hydrochloride ophthalmic, 0.5% drops (Bausch and Lomb, Bridgewater, NJ, USA) was introduced in each eye. The ILUX instrument delicately clamps on each eye lid, warming them for ∼90 s, and then applies gentle pressure on the eye lid to express the visible meibum magnified by ILUX. The expressate was collected with a platinum spatula and immediately dissolved in spectroscopy grade ultrapure chloroform in a glass microvial with a Teflon cap (Microliter Analytical Supplies Ind., Suwanee, GA, USA). The sample used was a pooled equiweight mixture of the MGS of four volunteers: two males (40 years old and 27 years old) and two females (41 years old and 28 years old). The MGS solution was stored at −80 °C until their use in experiments. The small sample size (i.e., the small number of volunteers from which the specimens were collected) is characteristic limitation of the biophysical studies of tear and meibomian samples. Typically, samples are either from a single donor, or pooled from a small number of volunteers (as done in the current study) [1,2,3,6,7,34,43,44,45,46]. Still, the studies of mutually independent teams collecting samples from vastly different (in terms of age, sex, ethnicity, etc.,) donors, confirmed the number of features characteristic for “healthy” meibum, i.e., the formation of noncollapsible viscoelastic duplex films of multilayer thickness at the air/water surface. This agreement between the results of different groups suggests that in spite of the small sample size of laboratory biophysical studies, the key meibum properties that are registered and analyzed are reproducible and representative.

The lipid profile of sesame oil (Rohto Pharmaceutical, Osaka, Japan) is summarized in Table 2 in the format required by the Japanese pharmacopoeia.

### 4.2. Methods

Surface pressure/area (π-A) isotherms were measured [6] by Langmuir surface balance µ Trough XS, area 135 cm^2^, volume 100 mL (Kibron, Helsinki, Finland), via the Wilhelmy wire probe (DyneProbe, Kibron Inc., Finland) method (instrumental accuracy 0.01 mN/m). Phosphate buffered saline (PBS, pH 7.4; Sigma-Aldrich, Saint Louis, MO, USA) was implemented as trough subphase. A micro-syringe (Hamilton Co., Reno, NV, USA) was used to uniformly spread human MGS, SO, or MGS/SO (as 1 mg/mL chloroform solution) over the air/saline surface. Prior measurement the trough is thoroughly rinsed with pure ethanol (Sigma-Aldrich, Saint Louis, MO, USA) and ultrapure water. The purity of PBS subphase is considered acceptable if upon full area compression the surface pressure remains <0.5 mN/m; if needed the surface is cleaned via vacuum aspirator. Typically, ≤25 µL of film-forming solution were spread as tiny 1–2 µL droplets across the entire trough surface. For calculation of the area per molecule, a mean molecular weight of Mw = 700 was assumed for MGS (which is an average of 650 for wax esters and 750 for cholesteryl esters and (*O*-acyl)-omega-hydroxy fatty acids) and Mw 870 for SO [34,47,48].

The trough was fitted under an acrylic cover to protect the surface from dust and to suppress the PBS subphase evaporation (90% relative humidity is maintained under the cover). After 15 min were provided for chloroform evaporation, film compression was initiated by two symmetrically moving barriers. Dynamic compression–expansion isocycling of the film area was performed at the maximum barrier’s rate (41.3 cm^2^/min), at which there was no leakage of the film. Five consecutive cycles were performed with each film studied. Typically, after the third cycle, the π(A) curves attained constant shape and those π(A) isotherms were collected and analyzed further. All isotherms were repeated at least in triplicate, and the difference between the repetitions was less than 2%. The experiments were performed at 35 °C, i.e., the physiological temperature of the ocular surface [49]. The films’ morphology was monitored by a Brewster angle microscope (UltraBAM, Accurion, Germany) with lateral resolution of ~2 µm.

The transient dilatational viscoelasticity of meibum films was also accessed by measurement of the surface pressure relaxation transients after a small rapid compression step was applied to the layers [45,50,51]. First, the film was compressed afnd allowed to equilibrate at initial surface pressure, π_0_ = 20 mN/m. Then, the film was instantaneously contracted with a small compression step, ∆A/Ao = 5% ± 1% (Ao is initial film area, and ∆A is area change). The ∆π relaxation transients were recorded and analyzed as explained further in the text.

## 5. Conclusions

Surface pressure (π)-area (A) isotherms and Brewster angle microscopy observations showed nonideal interactions at physiologically relevant packing densities and surface pressures, where SO aggregates with MGS and complements the NPL stratum of the meibomian duplex films. The analysis of stress relaxation transients with Kohlrausch–Williams–Watts equation revealed that the supplementation of fixed amount of MGS with excess lipids via SO altered the dilatational elasticity of the films as reflected by the increase of the exponent β. Thus, sesame oil with its unique combination of high oxidative stability, antioxidant content, and abundance of long polyunsaturated acyl chains might be a useful supplement to MGS layers in the formulations for the treatment of dry eye as well as for ophthalmic drug delivery [14].

## Figures and Tables

**Table 1:** Kohlrausch–Williams–Watts (KWW) equation parameters obtained from the fits of the stress relaxation transients (Figure 6, Left panel). For all the fits the adjusted R^2^ ≥ 0.98.

Film Composition	A, mN/m	τ, s	β	ΔπEQ, mN/m
MGS	3.539	90	0.486	1.80
SO	0.329	94.3	1.214	1.60
MGS/SO = 9/1	1.500	206.2	0.916	2.00
MGS/SO = 7/3	0.628	43	0.716	2.15
MGS/SO = 1/1	0.150	24.15	1.470	2.95

**Table 2:** Lipid profile of sesame oil presented in the format required by the Japanese pharmacopoeia. Less than 1% of the fatty acids are in free form. Rest are bound as acyl chains in triglycerides: 8% GS2U, 41% GSU2, and 51% GU3 respectively (here: G—glyceride backbone, U—unsaturated and S—saturated acyl chain respectively).

Composition of Sesame Oil	
Myristic acid	<1%
Palmitic acid	8–11%
Stearic acid	4–7%
Arachidic acid	0–1%
Palmitoleic acid	<1%
Oleic acid	35–43%
Linoleic acid	41–48%
Sesamin	1%
Sesamol	0.10%
Sesamolin	none

## Data Availability

The data presented in this study are available on request from the corresponding author. They may be made available to any researchers who wish to use them for non-commercial purposes, while preserving any necessary confidentiality and anonymity.

## Supplementary Materials

The following supporting information can be downloaded online, Supplementary materials: interactions between sesame oil (SO) and meibomian lipids (MGS) accessed via area increments

## Sample Availability

Sample of the sesame oil are available from the authors.

## References

[1] Willcox M.D.P., Argueso P., Georgiev G.A., Holopainen J.M., Laurie G.W., Millar T.J., Papas E.B., Rolland J.P., Schmidt T.A., Stahl U. (2017). TFOS DEWS II Tear Film Report. Ocul. Surf..

[2] Georgiev G.A., Eftimov P., Yokoi N. (2017). Structure-function relationship of tear film lipid layer: A contemporary perspective. Exp. Eye Res..

[3] Borchman D., Foulks G.N., Yappert M.C., Bell J., Wells E., Neravetla S., Greenstone V. (2011). Human meibum lipid conformation and thermodynamic changes with meibomian-gland dysfunction. Investig. Ophthalmol. Vis. Sci..

[4] McMahon A., Lu H., Butovich I.A. (2013). The spectrophotometric sulfo-phospho-vanillin assessment of total lipids in human meibomian gland secretions. Lipids.

[5] Butovich I.A. (2013). Tear film lipids. Exp. Eye Res..

[6] Georgiev G.A., Yokoi N., Ivanova S., Tonchev V., Nencheva Y., Krastev R. (2014). Surface relaxations as a tool to distinguish the dynamic interfacial properties of films formed by normal and diseased meibomian lipids. Soft Matter.

[7] Rosenfeld L., Cerretani C., Leiske D.L., Toney M.F., Radke C.J., Fuller G.G. (2013). Structural and rheological properties of meibomian lipid. Investig. Ophthalmol. Vis. Sci..

[8] McDonald M., Patel D.A., Keith M.S., Snedecor S.J. (2016). Economic and Humanistic Burden of Dry Eye Disease in Europe, North America, and Asia: A Systematic Literature Review. Ocul. Surf..

[9] Donthineni P.R., Kammari P., Shanbhag S.S., Singh V., Das A.V., Basu S. (2019). Incidence, demographics, types and risk factors of dry eye disease in India: Electronic medical records driven big data analytics report I. Ocul. Surf..

[10] Hsu E., Parthasarathy S. (2017). Anti-inflammatory and Antioxidant Effects of Sesame Oil on Atherosclerosis: A Descriptive Literature Review. Cureus.

[11] Martinchik A.N. (2011). Nutritional value of sesame seeds. Vopr. Pitan..

[12] Pathak N., Rai A.K., Kumari R., Bhat K.V. (2014). Value addition in sesame: A perspective on bioactive components for enhancing utility and profitability. Pharmacogn. Rev..

[13] Wan Y., Li H., Fu G., Chen X., Chen F., Xie M. (2015). The relationship of antioxidant components and antioxidant activity of sesame seed oil. J. Sci. Food Agric..

[14] Abou-Gharbia H.A., Shahidi F., Shehata A.A.Y., Youssef M.M. (1996). Oxidative stability of extracted sesame oil from raw and processed seeds. J. Food Lipids.

[15] Torrent-Burgués J. (2016). Langmuir films study on lipid-containing artificial tears. Colloids Surf. B Biointerfaces.

[16] Torrent-Burgués J., Hoyo J., Tzanov T. (2021). Lipid artificial tears at a mimetic ocular interface. Chem. Phys. Lipids.

[17] Caruso B., Maestri D.M., Perillo M.A. (2010). Phosphatidylcholine/vegetable oil pseudo-binary mixtures at the air-water interface: Predictive formulation of oil blends with selected surface behavior. Colloids Surf. B Biointerfaces.

[18] Smaby J.M., Baumann W.J., Brockman H.L. (1979). Lipid structure and the behavior of cholesteryl esters in monolayer and bulk phases. J. Lipid Res..

[19] Gershfeld N.L. (1976). Physical Chemistry of Lipid Films at Fluid Interfaces. Annu. Rev. Phys. Chem..

[20] Georgiev G.A., Kutsarova E., Jordanova A., Krastev R., Lalchev Z. (2010). Interactions of Meibomian gland secretion with polar lipids in Langmuir monolayers. Colloids Surf. B Biointerfaces.

[21] Georgiev G.A., Yokoi N., Nencheva Y., Peev N., Daull P. (2017). Surface Chemistry Interactions of Cationorm with Films by Human Meibum and Tear Film Compounds. Int. J. Mol. Sci..

[22] Mudgil P., Borchman D., Gerlach D., Yappert M.C. (2016). Sebum/Meibum Surface Film Interactions and Phase Transitional Differences. Investig. Ophthalmol. Vis. Sci..

[23] Mudgil P., Ramasubramanian A., Borchman D. (2020). Meibum lipid hydrocarbon chain branching and rheology after hematopoietic stem cell transplantation. Biochem. Biophys. Rep..

[24] Pogorzelski S.J., Watrobska-Swietlikowska D., Sznitowska M. (2012). Surface tensometry studies on formulations of surfactants with preservatives as a tool for antimicrobial drug protection characterization. J. Biophys. Chem..

[25] Davies J.T., Rideal E.K., Davies J.T., Rideal E.K. (1961). Chapter 5—Properties of Monolayers. Interfacial Phenomena.

[26] Vaidyanathan T.K., Vaidyanathan J. (2015). Validity of predictive models of stress relaxation in selected dental polymers. Dent. Mater. Off. Publ. Acad. Dent. Mater..

[27] Tsang K.Y., Ngai K.L. (1996). Relaxation in interacting arrays of oscillators. Phys. Rev. E.

[28] Avramov I. (1996). Kinetics of structural relaxation of glass-forming melts. Thermochim. Acta.

[29] Schmelzer J.W.P., Zanotto E.D., Avramov I., Fokin V.M. (2006). Stress development and relaxation during crystal growth in glass-forming liquids. J. Non-Cryst. Solids.

[30] Ewurum A., Ankem A., Georgiev G., Borchman D. (2021). A spectroscopic study of the composition and conformation of cholesteryl and wax esters purified from meibum. Chem. Phys. Lipids.

[31] Petrov P.G., Thompson J.M., Rahman I.B., Ellis R.E., Green E.M., Miano F., Winlove C.P. (2007). Two-dimensional order in mammalian pre-ocular tear film. Exp. Eye Res..

[32] Smaby J.M., Brockman H.L. (1981). Novel surface phase containing cholesteryl esters. 2. Nonequivalence of cholesteryl arachidonate and those with 18-carbon, cis-unsaturated acyl groups. Biochemistry.

[33] Mochizuki H., Yamada M., Hatou S., Tsubota K. (2009). Turnover rate of tear-film lipid layer determined by fluorophotometry. Br. J. Ophthalmol..

[34] Butovich I.A., Arciniega J.C., Wojtowicz J.C. (2010). Meibomian lipid films and the impact of temperature. Investig. Ophthalmol. Vis. Sci..

[35] Merker D.R., Daubert B.F. (1964). The Molecular Structure in Monolayers of Saturated Triglycerides on Water as Related to Three-Dimensional Polymorphic Forms. J. Am. Chem. Soc..

[36] Bursh T., Larsson K., Lundquist M. (1968). Polymorphism in monomolecular triglyceride films on water and formation of multimolecular films. Chem. Phys. Lipids.

[37] Cadena-Nava R.D., Martín-Mirones J.M., Vázquez-Martínez E.A., Roca J.A., Ruíz-García J. (2006). Direct observations of phase changes in Langmuir films of Cholesterol. Rev. Mex. De Fis..

[38] Sengupta A., Roychoudhury S.K. (1976). Triglyceride composition of Sesamum indicum seed oil. J. Sci. Food Agric..

[39] Singh C.P., Shah D.O. (1993). Oxidation of monoglyceride, diglyceride, and triglyceride monolayers by aqueous potassium permanganate solution. Colloids Surf. A Physicochem. Eng. Asp..

[40] Masayuki N., Noriaki F. (1974). The Formation and Collapse of Mixed Monolayer of Triolein and Tricaprylin. Bull. Chem. Soc. Jpn..

[41] Brown S.I., Dervichian D.G. (1969). The oils of the meibomian glands. Physical and surface characteristics. Arch. Ophthalmol..

[42] Mudgil P., Millar T.J. (2011). Surfactant properties of human meibomian lipids. Investig. Ophthalmol. Vis. Sci..

[43] Svitova T.F., Lin M.C. (2016). Dynamic interfacial properties of human tear-lipid films and their interactions with model-tear proteins in vitro. Adv. Colloid Interface Sci..

[44] Georgiev G.A., Yokoi N., Ivanova S., Dimitrov T., Andreev K., Krastev R., Lalchev Z. (2013). Surface chemistry study of the interactions of hyaluronic acid and benzalkonium chloride with meibomian and corneal cell lipids. Soft Matter.

[45] Nencheva Y., Ramasubramanian A., Eftimov P., Yokoi N., Borchman D., Georgiev G.A. (2018). Effects of Lipid Saturation on the Surface Properties of Human Meibum Films. Int. J. Mol. Sci..

[46] Arciniega J.C., Nadji E.J., Butovich I.A. (2011). Effects of free fatty acids on meibomian lipid films. Exp. Eye Res..

[47] Husain S., Sastry G.S.R., Raju N.P. (1991). Molecular weight averages as criteria for quality assessment of heated oils and fats. J. Am. Oil Chem. Soc..

[48] Rahman M.S., Hossain M.A., Ahmed G.M., Uddin M.M. (2007). Studies on the Characterization, Lipids and Glyceride Compositions of Sesame (Sesamum indicum linn.) Seed Oil. Bangladesh J. Sci. Ind. Res..

[49] Slettedal J.K., Ringvold A. (2015). Correlation between corneal and ambient temperature with particular focus on polar conditions. Acta Ophthalmol.

[50] Loglio G., Tesei U., Cini R. (1986). Viscoelastic dilatation processes of fluid/fluid interfaces: Time-domain representation. Colloid Polym. Sci..

[51] Monroy F., Ortega F., Rubio R.G. (1998). Dilatational rheology of insoluble polymer monolayers: Poly(vinylacetate). Phys. Rev. E.

