# Supplementary material for: Surface Chemistry Study of the Interactions of Sesame Oil with Meibomian Films
Source: Molecules. 2022 Jan 11;27(2):464. doi: 10.3390/molecules27020464 (PMC8777812; doi:10.3390/molecules27020464)
Supplement: Supplementary file 1 [file molecules-27-00464-s001.zip › molecules-1498084-supplementary.pdf]

Supplementary material: interactions between sesame oil (SO) and meibomian lipids (MGS) accessed via area increments

A commonly used alternative to Eq. (1) in the main text, is to evaluate the interactions between lipids in surface films via calculation of area increments with the help of Eq. (S.1)

$$\Delta A_{int} = A_{\pi,\ expl} - A_{\pi,\ ideal} \qquad (S.1)$$

Here

$$A_{\pi,\ ideal} = X_{MGS} \cdot (A_{MGS})_{\pi} + X_{SO} \cdot (A_{SO})_{\pi} \qquad (S.1a)$$

where X_{MGS} and X_{SO} are the molar fractions of the two components, and $(A_{MGS})_{\pi}$ and $(A_{SO})_{\pi}$ represent the surface areas of the “pure” components at a surface pressure π.

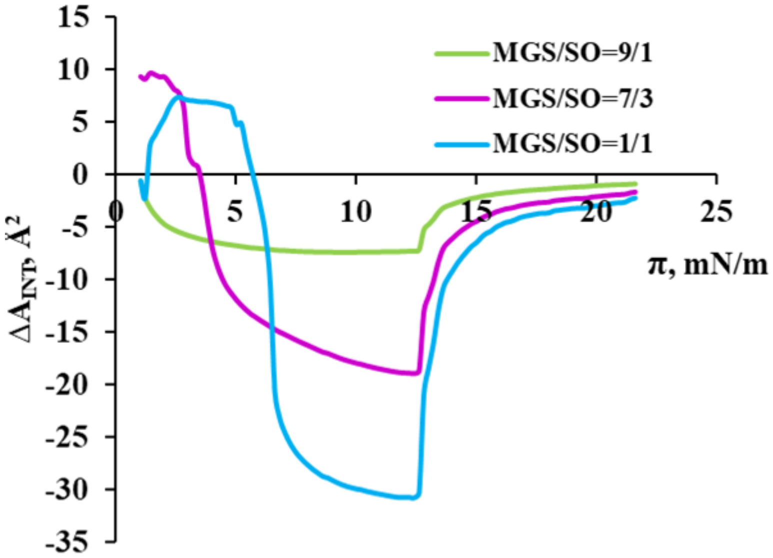

Fig. S1. Area increments calculated with Eq. (S1) to study the interaction of MGS and SO in surface films.

As explained in the main text due to the multilayer structure of the films Eq. (S.1) does not measure 2D intermolecular attraction or repulsion. Here the quantities indicate more complex transitions related to (i) aggregation of lipid molecules in thick surface domains or to (ii) the domains disaggregation at the interface. Thus:

- ΔA_{int} =0 denotes ideal behavior where the two compounds do not alter each other’s organization at the air/water surface
- An area expansion effect $\Delta A_{int} > 0$ may report for both, (i) repulsion between the lipid molecules in the plane of the film/water interface and/or (ii) disaggregation of the multilayer domains and redistribution of lipid molecules from the non-polar stratum to the aqueous interface
- An are reduction effect $\Delta A_{int} < 0$ may report for both, (i) aggregation between the lipid molecules in 2D at the film/water interface and/or (ii) increased aggregation of the molecules within the multilayer and redistribution of lipid molecules from the aqueous interface toward the film’ non-polar stratum facing the air. The formation of thicker brighter regions in the MGS/SO films visualized by the Brewster angle microscopy images (Fig. 2 in the main text) suggests that it is the latter phenomenon that takes place.

It can be seen that for all the MGS/SO compositions for most of the surface pressures $\Delta A_{int} < 0$ which similarly to the $\Delta\pi_{int}<0$ increments (Fig. 1 in the main text) indicates aggregation between SO and MGS due to preferable distribution of SO lipids in the nonpolar stratum of the MGS containing multilayers.

As mentioned in other studies (i.e. Fernandez-Botello et al., J. Phys. Chem. B 2008, 112, 13834; Pagano and Gershfeld, J Phys Chem. 1972, 76(9):1238-43.) surface films may display different sensitivity to the analysis with Eq. 1 and Eq. S1. In the current case the analysis with Eq. S1 is limited to the surface pressure of 21.7 mN/m (π reached upon compression of pure SO). In contrast Eq. (1) is applicable to higher surface pressures realized via compression of the surface films to very low apparent areas per molecule, and thus Eq. (1) is preferred for analysis of the MGS/SO interactions in the main text.
